# P-1719. Invasive Fusariosis in Immunocompromised Cancer Patients: Insights from a 17-Year Retrospective Cohort Study

**DOI:** 10.1093/ofid/ofaf695.1890

**Published:** 2026-01-11

**Authors:** David Kornblum, Jonathan D Andreadakis, Rod Quilitz, John Greene, Isis Lamphier, Ana Velez

**Affiliations:** USF, Clearwater, FL; Moffitt Cancer Center, Temple Terrace, Florida; Moffitt Cancer Center, Temple Terrace, Florida; Moffitt Cancer Center, Temple Terrace, Florida; Moffitt Cancer Center, Temple Terrace, Florida; University of South Florida, Tampa, Florida

## Abstract

**Background:**

Invasive *Fusarium* infections pose major therapeutic challenges and carry high mortality among immunocompromised cancer patients, particularly those with hematologic malignancies. Emerging antifungal resistance complicates management and underscores the need for updated clinical strategies.

**Table 1.**
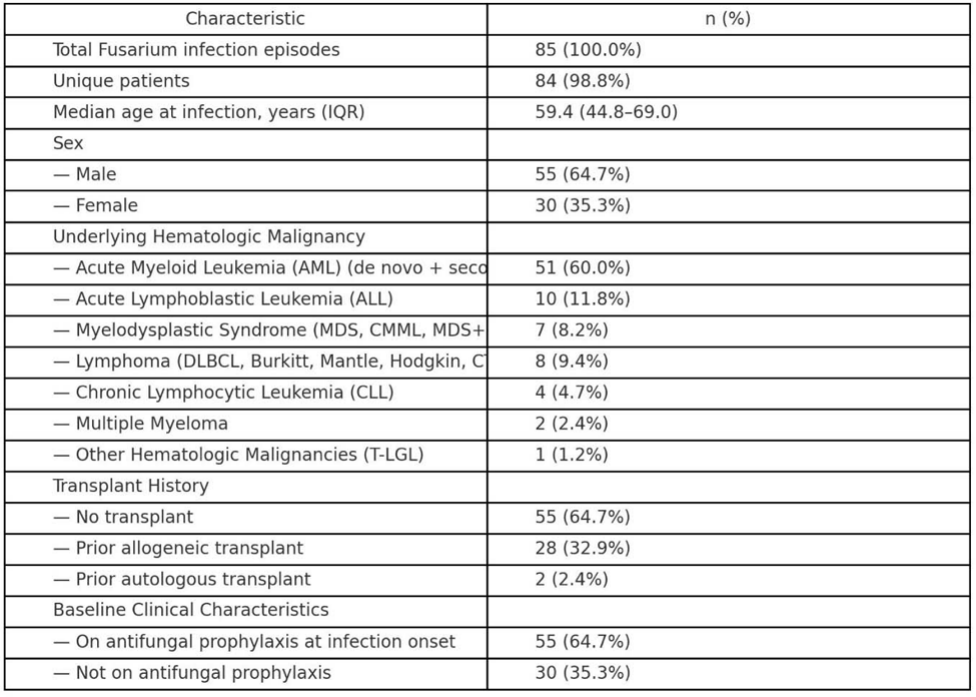
Baseline Characteristics of Patients with Fusarium Infection (N = 85 Episodes) Demographic and clinical features of 85 episodes of invasive Fusarium infection among 84 unique patients, including underlying hematologic malignancies, transplant status, and antifungal prophylaxis at the time of infection.

**Figure:**
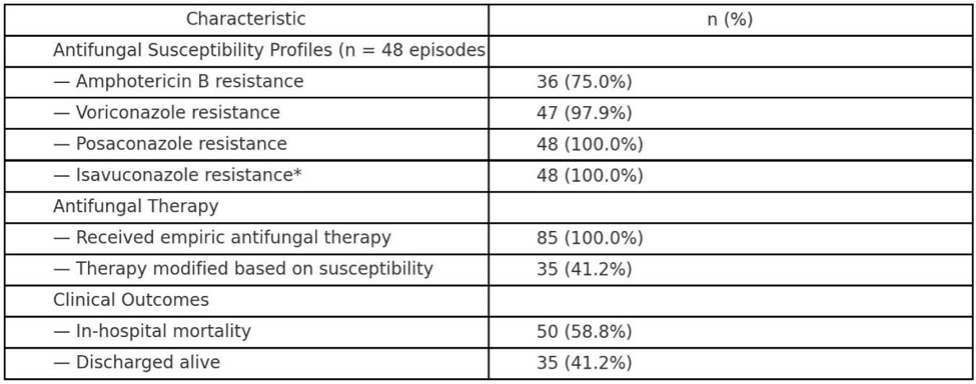
Table 2. Antifungal Susceptibility Profiles, Antifungal Therapy, and Clinical Outcomes (N = 85 Episodes) Summary of antifungal susceptibility results, empiric and directed therapy patterns, in-hospital outcomes, and associations between clinical factors and mortality or antifungal resistance.

**Methods:**

We conducted a retrospective cohort study of *Fusarium* infections at Moffitt Cancer Center from October 2008 to January 2025. A total of 85 patients with hematologic malignancies were included. Clinical, microbiological, and radiographic data, antifungal regimens, and outcomes were reviewed. Statistical analyses included descriptive statistics, Fisher’s exact tests, and odds ratios (OR) with 95% confidence intervals (CI).

**Table 3.**
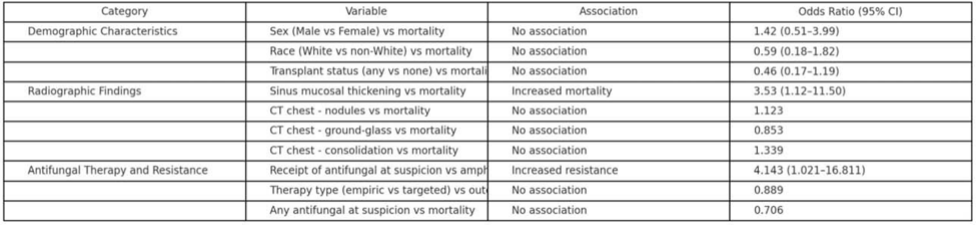
Associations Between Clinical Characteristics, Therapy, and Outcomes Univariate associations between patient characteristics, antifungal therapy variables, and outcomes including in-hospital mortality and amphotericin B resistance. Odds ratios (OR), 95% confidence intervals (CI), and p-values are shown for each comparison.

**Results:**

The cohort primarily included patients with AML (60.0% [51/85]), ALL (11.8% [10/85]), and other hematologic malignancies. Among 48 tested isolates, amphotericin B resistance was observed in 75.0% (36/48) and voriconazole resistance in 97.9% (47/48). Both posaconazole and isavuconazole resistance were universal (100%, 48/48). Empiric antifungal therapy was initiated in all episodes (85/85), with therapy modified in 41.2% (35/85) after susceptibility results. In-hospital mortality was 58.8% (50/85). Receipt of any antifungal agent at clinical suspicion was significantly associated with amphotericin B resistance (OR 4.143; 95% CI, 1.021–16.811; p=0.049). Amphotericin resistance rates appeared to increase over time.

**Conclusion:**

Invasive *Fusarium* infections are associated with poor outcomes and high antifungal resistance. Early empiric treatment may contribute to resistance emergence. Routine antifungal susceptibility testing is strongly encouraged to guide therapy and monitor local resistance trends. Rapid diagnostics, susceptibility-guided strategies, and access to novel agents like fosmanogepix are critical. Ongoing stewardship and multicenter studies are essential to improve outcomes.

**Disclosures:**

All Authors: No reported disclosures

